# Mr. Hume's Second Test for Arsenic

**Published:** 1810-08

**Authors:** Jos. Hume

**Affiliations:** Long Acre


### *To the Editors of the Medical and Physical Journal.*

Gentlemen,

Another very accurate and ready test for arsenic has just occurred to me, which, being easily performed, requiring neither expence, apparatus, nor a nice acquaintance with operative chemistry, may deserve the attention of your readers and the profession at large. It is, however, more suitable to detect the *dry* oxide itself than when in solution, and, therefore, as this poison is, I believe, always administered in powder when some fatal purpose is to be effected, the method I shall now suggest will consequently be found more practicable though not more certain than the former; but, at all events, one will serve to corroborate the other, for, when we can avail ourselves of an extended evidence in criminal conviction, nothing should be lost, nothing neglected.

From the difficult solubility of the white oxide, especially in cold fluids, we may in most cases obtain, from one source or other, a sufficient quantity for examination, for it is astonishing how very delicate these tests are, particularly the first, which will render the effect obvious when water is even slightly impregnated with the poison, so that a mere fractional part of a grain could not escape notice.

As an instance of this second process, let us take about a grain of the white powder, or suspected arsenic; mix this very intimately with two or three grains of nitrate of potash; potash; introduce this mixture, by the point of a penknife, into a small phial, in such a way, by inclining the phial horizontally, that the powder may remain undisturbed on the side or thinner part of the glass: now, apply the flame of a small lamp just under the powder, and presently, if there be arsenic, the nitrate will be decomposed, nitrous gas and nitrous acid evolved, and arseniate of potash will remain as the result.

I would prefer spirit of wine to oil, for the lamp, as it produces no smoke that can tinge the phial and intercept our seeing distinctly the yellow fumes peculiar to nitrous gas when in contact with the atmosphere. The acid vapour is easily recognised, either by its smell, by presenting the point of a feather imbued with solution of ammonia, or even by placing a morsel of moistened litmus paper within the phial and over the materials during this short operation.

As a farther support of this test, proceed thus with the new product, the arseniate of potash—fill the phial with warm rain water, that some of the salt may be dissolved; then, to the mere surface of the solution, present a piece of dry nitrate of silver (lunar caustic), and the peculiar dark brick-coloured precipitate, which I described in my last communication, will fully evince the fact, that arsenic had been administered.

Since either of the schemes I have proposed must soon become familiar and practicable, I trust your Correspondent, Mr. Jones, will, at his leisure, again submit some of the powder to trial; and I have no doubt he will readily favour us with a faithful report, whatever the result may be. I cannot quit my pen without acknowledging his kindness in offering the remainder of the powder for analysis; this I would willingly undertake, did I not implicitly depend upon his own accuracy, when assisted by the new means I have announced, as well as on his candour and liberality in confessing that the poison actually does exist in the same powder, should he succeed in the research.

I am, &c.

JOS. HUME.

*Long Acre, July* 13, 1810.

